# Need for a paradigm shift in soil-transmitted helminthiasis control: Targeting the right people, in the right place, and with the right drug(s)

**DOI:** 10.1371/journal.pntd.0012521

**Published:** 2024-10-21

**Authors:** Paul M. Emerson, Darin Evans, Matthew C. Freeman, Christy Hanson, Khumbo Kalua, Jennifer Keiser, Alejandro Krolewiecki, Lynn Leonard, Bruno Levecke, Sultani Matendechero, Arianna Rubin Means, Antonio Montresor, Denise Mupfasoni, Rachel L. Pullan, Lisa A. Rotondo, Mariana Stephens, Kristin M. Sullivan, Judd L. Walson, Tijana Williams, Jürg Utzinger

**Affiliations:** 1 Children Without Worms, The Task Force for Global Health, Decatur, Georgia, United States of America; 2 United States Agency for International Development, Washington, District of Columbia, United States of America; 3 Gangarosa Department of Environmental Health, Emory University, Atlanta, Georgia, United States of America; 4 Bill & Melinda Gates Foundation, Seattle, Washington, United States of America; 5 Blantyre Institute for Community Outreach, Blantyre, Malawi; 6 Swiss Tropical and Public Health Institute, Allschwil, Switzerland; 7 University of Basel, Basel, Switzerland; 8 Instituto de Investigaciones de Enfermedades Tropicales, Universidad Nacional de Salta/CONICET, Oran, Argentina; 9 Fundacion Mundo Sano, Buenos Aires, Argentina; 10 Johnson & Johnson, New Brunswick, New Jersey, United States of America; 11 Department of Translational Physiology, Infectiology and Public Health, Ghent University, Merelbeke, Belgium; 12 Kenya National Public Health Institute, Nairobi, Kenya; 13 Department of Global Health, University of Washington, Seattle, Washington, United States of America; 14 Ivo de Carneri Foundation, Milano, Italy; 15 Department of Control of Neglected Tropical Diseases, World Health Organization, Geneva, Switzerland; 16 London School of Hygiene & Tropical Medicine, London, United Kingdom; 17 RTI International, Washington, District of Columbia, United States of America; 18 Department of International Health, Bloomberg School of Public Health, Johns Hopkins University, Baltimore, Maryland, United States of America; 19 Liverpool School of Tropical Medicine, Liverpool, United Kingdom; Swiss Tropical and Public Health Institute, SWITZERLAND

## Background

“The worst form of inequality is to try to make unequal things equal”—Aristotle

Soil-transmitted helminthiasis (STH) is caused by intestinal parasites that require an obligate development period in the soil [1,2], predominantly roundworm (*Ascaris lumbricoides*), whipworm (*Trichuris trichiura*), and hookworms (*Ancylostoma duodenale* and *Necator americanus*), with the recent addition of threadworm (*Strongyloides stercoralis*). Historically, these parasites have been among the most common infections in humans, disproportionately affecting the world’s most disadvantaged and marginalized people.

The current approach to STH control and elimination as a public health problem is population-based, eschews adequate monitoring, assumes unlimited donated drugs which are not optimal against all STH species, and accepts massive programmatic inefficiencies. Hence, a new paradigm is needed. The burden of infection and morbidity due to these parasitic worms is rapidly declining, likely due to social and economic development and improvements in hygiene and living conditions, as well as the widespread distribution of donated or procured anthelmintic drugs. Despite this progress, there have been few changes in the structure of the global control program. The World Health Organization (WHO), which coordinates drug donations, reports that between 2010 and 2020, over 9 billion tablets were donated and distributed through STH-specific school-based programming, or to entire communities as part of the lymphatic filariasis (LF) elimination program [3]. In addition to the distributions reported by WHO, numerous other organizations, including UNICEF, have procured and distributed billions more deworming tablets. Collectively, there has been over a decade of extensive and continuous distribution of anthelmintic drugs reaching most at-risk populations (but crucially, not all).

The individual and population burden is unequal between localities, driven by the epidemiology of the disease. With the exception of *S*. *stercoralis* [4], soil-transmitted helminths do not multiply in the human host. As such, each adult parasitic worm is the result of an infection, meaning that prevalence, intensity of infection, and morbidity can be considered separate indicators. A single infection event does not lead to high worm burden or morbidity. Light infections may go unnoticed or cause mild symptoms, while tens or hundreds of worms (moderate-to-heavy intensity (MHI) infections) are associated with symptomatic disease. Heavy infections can result in intestinal obstruction (*A*. *lumbricoides*), rectal prolapse (*T*. *trichiura*), and pronounced anemia (hookworm) [5,6]. Between 2000 and 2019, disability-adjusted life years (DALYs) lost due to STH was reduced by 53%, from over 4.0 million to 1.9 million [7]. MHI infections are no longer ubiquitous in at-risk countries, and morbidity attributable to STH is becoming both less frequent and more focal [8,9].

Six ambitious targets and milestones were laid out in WHO’s 2030 Targets for Soil-Transmitted Helminthiases Control Programmes document (Table 1) [10]. To reach these goals, a new, targeted equity-based approach to deworming is required based on the relevant public health burden, historical drug access, and associated environmental and behavioral risk factors. WHO has shifted routine monitoring and reporting from an output indicator (i.e., coverage of deworming in the target populations) to add outcome and impact measures (i.e., prevalence of any infection and MHI infection). It is argued that this shift will allow programming to be delivered and measured against current epidemiology, not just population size.

**Table 1:** WHO 2030 targets and milestones for STH (source: https://iris.who.int/bitstream/handle/10665/330611/9789240000315-eng.pdf).

No.	Target	2030 Milestone
1	Achieve and maintain elimination of STH morbidity in preschool-age and school-age children by 2030	98 countries with <2% prevalence of children with STH of MHI infections
2	Reduce the number of tablets needed in preventive chemotherapy for STH	50% reduction
3	Increase domestic financial support for preventive chemotherapy for STH	25 countries deworming children with domestic funds
4	Establish an efficient STH control program in adolescent, pregnant, and lactating women of reproductive age	75% of women of reproductive age offered deworming medicine in endemic areas
5	Establish an efficient strongyloidiasis control program in school-age children	75% of children at risk of strongyloidiasis receiving ivermectin
6	Ensure universal access to at least basic sanitation and hygiene by 2030 in STH-endemic areas	Reduce open defecation to 0%

In many settings, all eligible residents in endemic countries may no longer warrant equal treatment based on current WHO guidelines. Populations in areas with high annual deworming coverage and good socioeconomic development need less treatment overall as compared to populations that have not had good coverage or those that remain impoverished. To maximize reductions in residual morbidity, programs should identify populations at highest risk of MHI infections and focus activities on those groups. This will involve the generation of reliable data and the redistribution of medicines and other resources based on need. This would be an equity-based approach compared to the current equality approach.

We propose 3 critical policy actions that constitute a paradigm shift in STH control and elimination that will increase eligible population reach and perhaps interrupt transmission and eliminate the parasites: (i) target deworming drugs to the right people, in the right place, in the right quantity; (ii) use the right drug(s) for the prevalent species; and (iii) increase coordination, accountability, and country ownership. In this new paradigm, global control of STH can be more equitable, more effective, and more cost-efficient.

### Policy action 1: Target deworming drugs to the right people, in the right place, in the right quantity

*“Equity’s role is to prevent the law from adhering too rigidly to its own rules and principles when those rules and principles produce injustice”*—*Aristotle*

With the first large donation of anthelmintic drugs for STH and morbidity control as the primary objective, health and education ministries sought to scale up access as quickly as possible, for as little cost as possible. The strategy adopted in many settings was school-based deworming administered by teachers, with the frequency of deworming determined by a baseline threshold. District-level prevalence was often established from aggregated available national data and erred toward increased access since donated high-quality drugs were extremely safe and offered in a one-tablet single oral dose. Deworming campaigns have also been integrated with other neglected tropical disease (NTD) control programs and delivered to whole communities, but stand-alone administration is usually school based. The epidemiologic profile of endemic countries is likely to have changed considerably since programs were initiated, and there is an urgent need for surveys with sufficient precision to target those still at risk. To improve targeting of deworming drugs based on empirical data and epidemiological evidence, this may mean moving away from district-level implementation units to smaller ones that better delineate areas of ongoing transmission and targeting high-risk populations within geographies. This would require a more considered sampling approach tailored to the setting and decisions to be made.

With an anticipated reduction in prevalence and intensity of infection as the programs reach high population and geographic coverage, WHO guidelines call for impact surveys after ≥5 years of regular deworming [10]. As of 2019, only 15/102 (approximately 15%) of endemic countries have been able to collect these crucial monitoring data [11]. As a result, drug requests based on population rather than current epidemiology continue without being contingent on evidence of morbidity. Where programs have conducted parasitologic surveys, STH prevalence and intensity have been dramatically reduced, and the morbidity target met [2,9,12]. Conversely, where control programs have failed to reach adequate geographic and population coverage, there are likely hot spots with high parasite burdens and risk of morbidity. Continuing with the status quo of population-driven, rather than prevalence and morbidity-driven, programming fails to adequately account for impact of the program and increasingly different epidemiologic situations of prevalence and intensity following years of deworming.

Population-based programming may also exacerbate the challenges associated with the limited resources available for STH control programs. Despite being cost-effective [13], deworming programs place significant demands on governments and communities regarding time, energy, attention, and resources. An epidemiologic-informed approach will help allocate scarce resources toward individuals who still require treatment and away from those who are no longer at risk. While funding for monitoring STH control programs is typically limited, investing in field-based epidemiologic surveys is crucial after ≥5 years of successful deworming to target treatment. Since the risk of intestinal worms is environmentally linked, geostatistical modeling approaches can be used to guide programs toward high-risk areas and to make decisions on treatment frequency [14]. Where the WHO NTD Road Map target has not been reached, and people are still at risk of parasite-related morbidity, STH control programs must move beyond the school and embrace context-specific innovative delivery platforms and a comprehensive strategy that leaves no one behind. These may include integration with other mass drug administration programs, antenatal clinics, immunization programs, or other population-based public health programs.

### Policy action 2: Use the right drug(s) for the prevalent parasite species

Despite over a decade of deworming efforts, certain geographic areas still have MHI infection prevalence among children that surpasses the WHO-specified threshold of 2% [15]. This can, in part, be attributed to high baseline prevalence and programmatic issues such as low treatment or geographic coverage; however, the choice of therapeutic regimen might also contribute to the persistence of these infections [16].

Large-scale deworming programs are typically based on albendazole (400 mg) or mebendazole (500 mg) monotherapy. This is not because these regimens are the most efficacious or the best choice in clinical practice but because they are safe and easy to administer (a standardized single oral dose) and are suited to scale up in large populations because of the donation programs. While monotherapy albendazole and mebendazole are highly efficacious against *A*. *lumbricoides* (egg reduction rate (ERR): >95%), they are considerably less efficacious against *T*. *trichiura* (ERR: >50%). Similarly, the drugs are efficacious but suboptimal for hookworm (albendazole ERR: >90%, mebendazole ERR: >70%) [16]. The scientific evidence that there are more effective therapeutic regimens using single or combination therapy is compelling. However, shifting programs away from less-efficacious but free drugs to the most efficacious drugs requires a paradigm shift in thinking [17,18]. An exception is the combination therapy of albendazole plus ivermectin, which has already been safely distributed in areas where STH was co-endemic with LF. This combination was recently added to the WHO Model Lists of Essential Medicines for treating STH [19].

In addition to the superior therapeutic efficacy for *T*. *trichiura* and hookworm [17], and the long-term programmatic impact on *T*. *trichiura* infections [20], this combination therapy has several advantages over monotherapy. Firstly, it has intrinsic efficacy in controlling *S*. *stercoralis*, and hence, upscaling this combination therapy for STH control contributes to WHO targets 1 and 4 (Table 1). Secondly, it has been shown in veterinary medicine that combination therapy slows the emergence of anthelmintic resistance [21]. Thirdly, ivermectin can reduce other NTDs, including scabies, ectoparasites, and mosquito-borne diseases [22]. The administration of albendazole plus ivermectin combination therapy is more challenging than monotherapy as the ivermectin dose can be more than one tablet, based on height. Research is being conducted to evaluate a single fixed dose albendazole/ivermectin co-formulated tablet [23].

Targeting the right parasite species with the right drug(s) is crucial for optimally treating STH and achieving 2030 milestones. Novel treatments are currently being developed, with emodepside being the front-runner [17]. Yet, we do not have to wait for these compounds to reach the market: in intervention areas where morbidity from any species is low or where *A*. *lumbricoides* is the dominant species, monotherapy with either albendazole or mebendazole is an optimal use of resources; where hookworm is prevalent, albendazole should be used; and where *T*. *trichiura* is the predominant species, programs should use albendazole plus ivermectin. The time is now to reset the strategy to sustainably focus on offering the remaining affected people deworming drugs that are locally effective against the parasites causing morbidity.

### Policy action 3: Increase coordination, accountability, and country ownership

WHO has provided guidance for national deworming programs, while pharmaceutical companies and other agencies have donated billions of deworming tablets, and national programs and their partners have mobilized to distribute them, all greatly reducing morbidity attributable to intestinal worms [24]. The strategy has been based on assumptions: universally high prevalence and MHI infections across endemic areas and the continuation of drug donations in perpetuity. Neither of these assumptions are still valid. The future will need to be different from the past, with donated drugs targeted to populations at risk in lower-income countries, while middle-income countries reduce the frequency of deworming where morbidity is controlled and purchase medicines for areas where population-level deworming is still indicated. In an environment of increasing country ownership and declining donated resources, allocating those resources should be optimized. Treatment and monitoring, and evaluation guidelines exist but are not always applied. To optimize the programs, there needs to be a transparent and defensible allocation of donated drugs and financial resources. With a restated alignment to the WHO NTD Road Map goal of eliminating morbidity from STH (defined as <2% MHI infection prevalence among children), stakeholders must develop a coordinated strategy to achieve and measure it. This will need to include an accountability/sustainability framework with consensus to apply it. With such a coordinated strategy, delivery and program monitoring objectives can be developed for each partner that considers the “who, where, what, and how” of program delivery. “Who” are the target groups at risk of morbidity? “Where” are they, “what” are the dominant parasite species, and what are the best treatment regimens for them? With this knowledge at hand, the “how” to reach the right people, in the right place, with the right drug(s) with high coverage and minimal cost can be addressed.

Global and national-level coordination between partners will improve efficiency, free up precious time for health care personnel, save money, reduce inadvertent duplication of effort, and allow integration of deworming not only with other NTD programs such as LF, schistosomiasis, and trachoma but also with immunization, maternal child health, and other primary healthcare activities. At this pivotal point in the global control program, improving collaboration and accountability within the global STH control program is crucial for continued success and investment. By fostering cooperation, accountability, and responsible resource allocation, we can address the persistent challenges faced by control efforts more effectively. To achieve this level of coordination, we must bring together key actors to reinforce commitments and create a unified strategy for STH control within the broader context of integrated healthcare initiatives.

## Conclusions

Despite decades of debate regarding the most effective approach to control STH globally, few changes have occurred in the structure of the global control program. Multiple global deworming campaigns, multi-stakeholder declarations, regional and national meetings, and research have all suggested that innovative, targeted approaches are needed to complement school-based deworming. Huge progress has been made, but despite this, millions of people remain at risk of morbidity from intestinal parasite infections. While some might argue that nothing presented here is new, our point is that the time and context have changed. Through the World Health Assembly, endemic countries have endorsed the WHO NTD Road Map goal of eliminating morbidity from STH by 2030 and are poised to implement it.

Deworming as a public health intervention is an effective preventive therapy for people living in endemic areas of the world. For deworming to remain effective, it is time to update policies and practices such as the guidelines for coadministration of preventive chemotherapy for NTDs and ensure that current epidemiologic trends are incorporated into program design and planning. We can only ensure that the right people can benefit in the right places by carefully revising our approach. The global STH—and indeed the global NTD—community must commit to working together to make this happen.
